# Creatine monohydrate supplementation strategies on body composition and water distribution in female recreational athletes

**DOI:** 10.1080/15502783.2025.2533668

**Published:** 2025-07-20

**Authors:** Isaac H. Avon, Kyle S. Levers, Natalia Wasilcyzk, Eden Glick, Eleanor U. Flacke, Anneliese Silverman, Payton Lynch, Alex Rainey, Henry Ball, Ashleigh Sorokin, Andrew M. Stranieri, Yichen Jin, Todd H. Miller

**Affiliations:** Department of Exercise & Nutrition Sciences, George Washington University, Washington, DC, USA

**Keywords:** Creatine supplementation, body composition, water distribution, female athletes, bioelectrical impedance analysis, dual-energy x-ray absorptiometry

## Abstract

**Background:**

Creatine monohydrate (CrM) supplementation has been reported to increase cellular water retention (WR) and alter body composition (BC) in anaerobically-training cohorts, but research in female athletes is limited. The study purpose was to examine the impact of two CrM supplementation strategies on BC and WR in female recreational athletes.

**Methods:**

Eleven female recreational athletes (Mean ± SD: 19.90 ± 1.51yrs, 163.88 ± 7.05 cm, 64.83 ± 9.43kg, 29.55 ± 6.03 %BF) participated in three testing sessions across the 14-day supplementation timeframe: 0-day (0D), 7-day (7D), and 14-day (14D). Participating subjects met standards prior to admittance. Subjects were randomly assigned to three supplement groups in a double-blind manner: loading (CF-L, 20g CrM + 15g Maltodextrin), maintenance (CF-M, 5g CrM+30g Maltodextrin), and placebo (PLA, 35g Maltodextrin). Subjects ingested the supplement 1x/d before noon and maintained standardized conditions (fasted (>6h), caffeine-free (>12h), exercise (>24h) before testing with study period alcohol abstinence. BC and WR were assessed via DXA and BIA, respectively, with pretest urine specific gravity hydration confirmation. Two-way repeated measures ANOVAs with Tukey HSD post hoc testing evaluated metrics across groups and time.

**Results:**

Despite different CrM supplementation strategies, body mass (BM, p=0.109, ηp^2^=0.744), body fat % (BFp, p=0.493, ηp^2^=0.162), fat mass (FM, p=0.411, ηp^2^=0.120), lean soft tissue (LM, p=0.833, ηp^2^=0.045), and fat-free mass (FFM, p=0.844, ηp^2^=0.042) did not change. Intracellular water (ICW, p=0.189, ηp^2^=0.471), extracellular water (ECW, p=0.170, ηp^2^=0.530), and total body water (TBW, p=0.181, ηp^2^=0.496) remained unaffected, while ECW/TBW demonstrated 0D-14D modification (p=0.004, ηp^2^=0.595). CF-L decreased ECW/TBW (Δ-1.35%, p=0.004) 0D-14D, while CF-M (Δ-0.45%, p=0.939) and PLA (Δ0.74%, p=0.451) facilitated insignificant ratio alteration.

**Conclusion:**

Fourteen-d CrM supplementation in female recreational athletes during an active training period did not facilitate significant tissue changes. However, 14-d CrM-L lowered ECW/TCW, facilitating greater intracellular water and nutrient retention compared to CrM-M strategies. Aesthetic or weight class-based female athlete populations may derive metabolic/neuromuscular benefits from moderate duration CrM loading.

